# Catalytic Dehydration of Glycerol to Acrolein in a Two-Zone Fluidized Bed Reactor

**DOI:** 10.3389/fchem.2019.00127

**Published:** 2019-03-14

**Authors:** Benjamin Katryniok, Roger Meléndez, Virginie Bellière-Baca, Patrick Rey, Franck Dumeignil, Nouria Fatah, Sébastien Paul

**Affiliations:** ^1^CNRS, Centrale Lille, ENSCL, Univ. Lille, Univ. Artois, UMR 8181 - UCCS - Unité de Catalyse et Chimie du Solide, Lille, France; ^2^ADISSEO France SAS, Antony, France

**Keywords:** glycerol, dehydration, acrolein, heterogeneous catalysis, two-zone fluidized bed reactor

## Abstract

The gas-phase catalytic dehydration of glycerol to acrolein was carried out in a Two-Zone Fluidized-Bed Reactor (TZFBR) using a 20 wt. % phosphotungstic acid (H_3_PW_12_O_40_) catalyst supported on CARIACT-Q10 commercial silica. In the first step, a hydrodynamic study of the reactor was performed. A quality of fluidization of more than 80% was obtained. In the second step, the mechanical stability of the catalyst was studied. It was found that only the external layer of active phase is eliminated under the conditions of operation whereas the global composition of the catalyst was not significantly affected after 44 h of fluidization. Finally, in a third step, the influence of the main operating parameters on the overall catalytic performances (glycerol/oxygen molar ratio and relative volumes of the reaction and regeneration zones) was investigated, showing notably the importance of the O_2_/glycerol ratio, resulting in an inverse trend between conversion and selectivity. Increasing O_2_/glycerol ratio led to higher conversion (lower coke deposit as shown by TGA analysis), but to the detriment of the selectivity to acrolein, supposedly due to the presence of O_2_ in the reaction zone causing the degradation of glycerol and acrolein.

## Introduction

The glut of glycerol issued from the industrial process of vegetable oil transesterification for the production of biodiesel has led to numerous studies on the gas-phase dehydration of glycerol to acrolein in the past 10 years (Katryniok et al., 2009, 2010, 2013; Voegele, 2011). This reaction is generally catalyzed by solids exhibiting acid properties, which can be classified in three distinct groups: (i) supported Keggin-type heteropolyacids (HPA), (ii) zeolites, and (iii) metal oxides such as WO_3_-ZrO_2_ or Nb_2_O_5_ (Katryniok et al., 2010). In the first and second groups, the catalysts are efficient during the first hours under stream but they suffer from rapid deactivation because of coke formation blocking the access of reactants to the acid active sites. For example, the best Keggin-type HPA catalyst studied by Alhanash et al. (2010) initially reached a glycerol conversion of 100% and an acrolein selectivity of 98%, but the performances rapidly decreased after 6 h under stream (loss of roughly 40% of conversion). In the third group, the rate of deactivation by coking is generally lower and good performances can be kept at least during 200 h under stream. However, the performances—and especially the initial performances—are generally lower (acrolein yield in the range 60–70%) than those observed on the catalysts of groups 1 and 2 (acrolein yield in the range 85–95% or even more). As an illustration, the zirconium-niobium mixed oxides studied by Lauriol-Garbay et al. still exhibited 82% conversion of glycerol and a selectivity to acrolein of 72 % after 177 h on stream, while, initially, full glycerol conversion, and roughly 70% of selectivity to acrolein were reached (Lauriol-Garbey et al., 2011). Hence, irrespective of the type of acid catalyst used to carry out the glycerol dehydration reaction, the deactivation by coking unavoidably occurs. This notably prevented the commercialization of the acrolein production from glycerol until now. Indeed, the unavoidable deactivation phenomenon impedes the development of a competitive process which would be necessary for cost-effective industrial exploitation.

Then, in parallel to the optimization of the heterogeneous catalyst properties, an adequate regeneration technology of the catalyst would be highly desirable. Some groups worked on that aspect, and Table 1 summarizes the three main regeneration technologies proposed so far.

**Table 1:** Main regeneration technologies proposed to extend the life of glycerol dehydration catalysts.

**Regeneration technology**	**Main disadvantages**
Co-feeding of oxygen or hydrogen	Risk of explosive conditions Formation of side products
Cyclic regeneration with oxygen or air under flow or by pulse injection	Loss of productivity High capital costs (spare reactor)
Continuous regeneration by FCC-like technology (2 fluidized-bed in series)	Attrition of catalyst particles High capital expenditure

The first approach consists in co-feeding oxygen (or hydrogen) in the reactor to eliminate the coke immediately after its formation on the catalyst. In this case, continuous regeneration of the catalyst is achieved, but the risk of yielding explosive conditions in the reactor and the possible formation of side products issued from subsequent reactions of acrolein or of other products with oxygen (or hydrogen) are serious disadvantages which acted as barriers to wider application of this solution.

To isolate glycerol and acrolein from oxygen (or hydrogen), a cyclic regeneration strategy (possibly using intermediate purges with an inert gas to avoid any risk of yielding explosive atmospheres), sometimes called “semi-regenerative process,” can be chosen. However, in this case, the process productivity is low because of the frequent need to stop the reaction for regenerating the catalyst. A solution could be to work with a two-reactor configuration in the so-called switch mode (one reactor being in operation when the second one is in the regeneration mode), but then the capital expenditure of the process is substantially higher.

Another solution was proposed to avoid the interruption of the production of acrolein. It consists in using a fluidized catalytic cracking (FCC)-like process often called “moving-bed reactor.” In such a configuration, two separate reactors placed in a loop configuration are used for the reaction and the regeneration steps, respectively. A continuous regeneration is carried out in this case. It was first employed by Corma et al. (2008) and O'Connor et al. (2008) with zeolite-based catalysts. The authors used a MicroDowner reactor to simulate the industrial fluid catalytic cracking process. Temperatures were in the range of 290–650°C with contact times of 0.5–30 s. The highest yields in acrolein were relatively low (around 55 to 60%) at 350°C, using a ZSM-5 catalyst, at low contact times of 0.5–2 s, which corresponds to WHSVs in the range of 300 to 1,300 h^−1^. The disadvantage of a conventional FCC-like reactor is the complexity of the system, consisting of two independent riser and regeneration reactors, thus demanding high investment costs.

**Figure 1:**
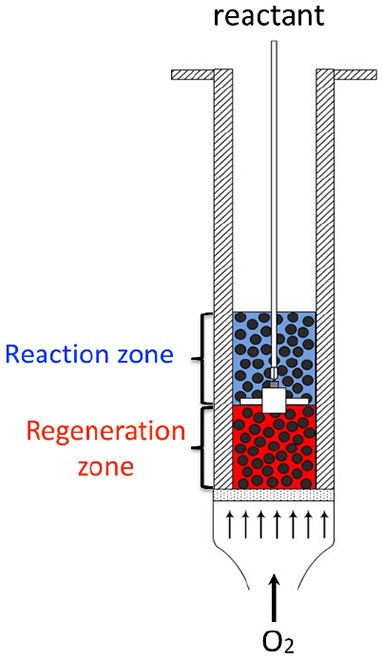
Schematics of the Two-Zone Fluidized-Bed Reactor (TZFBR) concept.

Finally, in the 80s, a new reactor concept was developed by Wheelock where both the reaction and the regeneration take place in the same volume, thus involving reduced capital expenditure from the resulting process (Wheelock, 1997). The reactor can roughly be described as a single fluidized bed, where the reactant is injected in the middle of the bed via a nozzle (Figure 1). The regenerating agent (oxygen or hydrogen) is injected in the fluidized bed at the bottom of the reactor. Thus, two zones can be distinguished: the bottom zone, where the catalyst is only in contact with the regenerating agent, and the top zone, where the catalyst is in contact with the reactant gas. The corresponding concept—called Two-Zone Fluidized-Bed Reactor (TZFBR)—was applied for regeneration of an ethanol-reforming coked catalyst, (Pérez-Moreno et al., 2012) but also and mainly for decoupling the oxidation and reduction steps of catalyzed reactions following a Mars and Van Krevelen mechanism [i.e., styrene synthesis, (Cocco and Castor, 2001) oxidative dehydrogenation of butane and propane] (Soler et al., 1999; Gascón et al., 2005; Lobera et al., 2009).

In our previous studies, we have shown that Keggin-type heteropolyacids supported on silica can give excellent initial performances in glycerol dehydration to acrolein with yields larger than 90%, but also suffer from fast deactivation by coking (Katryniok et al., 2012). This makes this kind of catalyst an excellent candidate to be studied in a TZFBR. The idea is to check if it would be possible to maintain the initial excellent performances by continuously eliminating the coke formed on the catalyst surface and porosity during the reaction. Therefore, in the present work, a TZFBR was used to carry out the glycerol dehydration to acrolein catalyzed by a Keggin-type heteropolyacid (20 wt.% of phosphotungstic acid, H_3_PW_12_O_40_) supported on CARIACT-Q10 commercial silica. The TZFBR design made it possible to simultaneously conduct the two steps of the process, namely the reaction of glycerol dehydration and the regeneration by oxygen of the coked catalyst, in a single reactor. The feasibility of the continuous acrolein production from glycerol was therefore shown for the first time using this reactor technology (Pariente et al., 2012).

## Experimental

**Figure 2:**
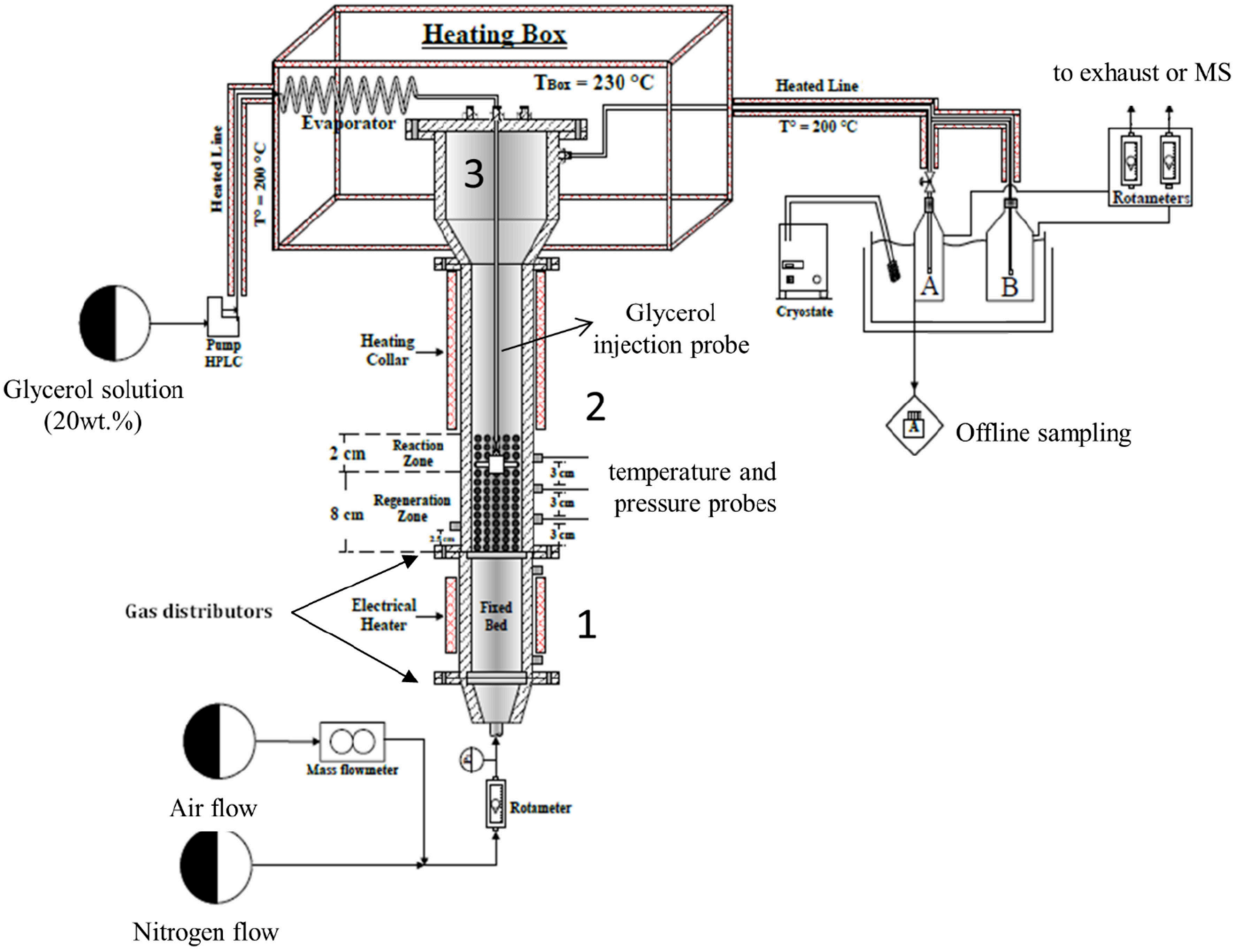
Schematics of the TZFBR setup.

A schematic diagram of the experimental set-up (TZFBR) used in this work is illustrated in Figure 2. The fixed bed (Zone 1), the fluidized bed reactor (Zone 2), and the disengagement zone (Zone 3) were all made of stainless steel 304. The fixed bed had an inner diameter of 50 mm and a total height of 225 mm. The main objective of this fixed bed was to efficiently pre-heat the fluidization gas before it entered the fluidization column. Therefore, it was filled with SiC beads (2 mm diameter) to improve heat transfer. A porous plate 70 mm in diameter and 2 mm thick made of Inconel and presenting a pore size of 40–50 μm was used as a gas distributor for Zones 1 and 2. The fluidization column had a 50 mm inner diameter and a total height of 700 mm. It was equipped with three thermocouples in order to check the temperature homogeneity along the fluidized-bed during the reaction. A pressure probe was installed at a distance of 2.5 cm over the distributor plate (bottom of the fluidization column). The pressure probe was connected to a system of U-tube manometers to determine the pressure drop (Δ*P*) through the fluidized bed with reference to the atmospheric pressure. The disengagement zone (Zone 3) had an internal diameter of 85 mm in the upper section, of 50 mm in the bottom section, and a total height of 275 mm total. This disengagement zone enabled the reduction of the gas velocity and prevented the entrainment of most of the catalyst particles by the gas flow. However, for the attrition test (see below for description) a cyclone (not shown on Figure 2) with an internal diameter of 15 mm and a total height of 60 mm was installed at the outlet of Zone 3. Its main function was to recover any small particles which could be possibly formed by attrition (erosion) of the catalyst and then subsequently dragged in the gas flow. The cyclone was removed during catalytic experiments to avoid acrolein condensation in the cold spot, as the attrition test showed that the formation of fine particles was negligible (see below).

Finally, a feed tube equipped with a nozzle was installed inside the fluidization column. The feed tube was used to inject the evaporated glycerol aqueous solution directly in the catalytic fluidized bed. The feed tube position could be changed vertically to increase or decrease the volumes of the reaction and regeneration zones correspondingly.

The reaction conditions used were as follows: An aqueous solution of 20 wt.% glycerol was fed with a volumetric flow of 0.5 mL/min by a HPLC pump (Gilson 305). The feed was progressively pre-heated at 200°C before entering into an evaporator at 230°C in order to ensure complete evaporation of the solution. A mixture of air and nitrogen was used as fluidization gas. It contained 0, 1, 3, 6, or 21 mol.% of oxygen and its flow was monitored by a Q-Flow rotameter from Voegtlin. The air flowrate was set by a Brooks mass flow controller.

In a first step of the study, the quality of the fluidization was checked at 275°C using 96 g of catalyst, which corresponded to a fixed bed height of 10 cm. Moreover, an attrition test was also performed at 275°C during 44 h. Air was employed as a fluidization gas to conduct these experiments, which were carried out in the absence of glycerol.

In a second step, catalytic tests were performed using 96 g of catalyst. The nozzle for the injection of the glycerol solution was placed at 5 or 8 cm from the distributor, thus modifying the volumes of the reaction and regeneration zones. The reaction temperature was 275°C. When the desired temperature was reached (after around 4 h because of the thermal inertia of the TZFBR), the glycerol feed started to be injected in the fluidized-bed reactor via the inlet nozzle (this is the t0 considered in **Figures 7–10**). A further period of 4 h was required for stabilizing the glycerol feed in the TZFBR. This step, which was carried out using pure nitrogen as fluidizing gas, is important to enable formation of a layer of coke on the catalyst before starting the oxygen injection in the system. In the absence of coke, the oxygen would react with glycerol or acrolein in the upper part of the fluidized bed, notably leading to the formation of byproducts.

Then, after the 4 h of stabilization, the fluidizing nitrogen flow was mixed with 2.7, 3.6, 11, or 21 NL/h of air in order to obtain an oxygen/glycerol molar ratio of 0.3, 0.4, 1.3, or 2.4, respectively. The catalytic performances were measured over 3 days (55 h under stream in total), as follows: The first day, after glycerol feed stabilization, two cold traps were collected (between 4 and 6 h under stream) and then the test was left to run overnight. The second day (between 20 and 30 h under stream) 5 cold-traps were collected and analyzed. Finally, the third day (between 45 and 55 h under stream) 4 cold-traps were collected and analyzed and then the test was stopped.

The products and the non-reacted glycerol were recovered using a cold trap at the outlet of the set-up. They were analyzed off-line on an Agilent 5890 gas chromatograph equipped with a FID detector and a CB-WAX column (L: 30 m, ID: 53 μm). Acrolein was the main product found in the cold trap, with very small traces of hydroxyacetone, acrylic acid, acetaldehyde and propionaldehyde, which were not taken into account in the calculation of the carbon balance. In the operating conditions where the glycerol was not fully converted, cyclic acetals resulting from the reaction between glycerol and acrolein were also detected but not quantified and hence were not included in the carbon balance either. Finally, CO_2_ was also formed but, because of its high dilution in the fluidizing gas, it was impossible to quantify it and consider it for the carbon balance calculation.

The glycerol conversion (*X*), the product's selectivities (*S*), and yields (*Y*) and the carbon balance were calculated as follows:

(1) $$\begin{array}{l} X=1-\frac{{ṅ}_{glycerol\left(0\right)}}{{ṅ}_{glycerol\left(i\right)}} \end{array}$$

(2) $$\begin{array}{l} S=\left(\frac{\gamma_{i}\;}{\gamma_{glycerol}}\right)*\left(\frac{{ṅ}_{i}}{{ṅ}_{glycerol\left(i\right)}-{ṅ}_{glycerol\left(o\right)}}\right)x100 \end{array}$$

(3) $$\begin{array}{l} Y=S\;*\;X \end{array}$$

(4) $$\begin{array}{l} BC=\frac{\sum \gamma_{i}*{ṅ}_{i}+\gamma_{glycerol}\;*\;{ṅ}_{glycerol\left(o\right)}}{\gamma_{glycerol}*{ṅ}_{glycerol\left(i\right)}} \end{array}$$

Where γ_*i*_ is the number of carbon atoms in the product *i*, γ_*glycerol*_ the number of carbon atoms in glycerol (i.e., 3) and ṅ_*i*_ and ṅ_*glycerol*_ the molar flowrates of product *i* and of glycerol, respectively. *(o)* and *(i)* refer to the outlet and the inlet, respectively.

Raman spectroscopy was carried out on an ISA Dilor-Jobin Yvon-SPEX Horiba instrument at room temperature using a laser beam of 532 nm, a confocal hole size of 50 μm and acquisition times of 30 s and 200 s for fresh and fluidized catalyst, respectively, and 60 s for the catalyst after reaction. The analysis was performed from 200 to 1,600 cm^−1^.

The ICP-MS analyses were performed using a Thermo-Fisher X7 ICP-MS to determine the experimental quantity of silicon and tungsten of the catalyst after fluidization.

Scanning electron microscopy (SEM) images were taken with a Philips SEM 505 scanning microscope equipped with an EDX Philips 505 microprobe at 5 and 20 keV.

TGA and DSC analyses of used and of fresh catalysts were carried out under air (flow rate 100 mL/min). The samples were heated from room temperature to 550°C at a temperature ramp of 3°C/min in a Labsys TGA-DTA 1,600 apparatus.

The surface areas, pore sizes, and total pore volumes of the catalysts before and after reaction were measured by N_2_ adsorption-desorption. A Micromeritics ASAP 2000 was employed to carry out these experiments. The samples were degassed at 130°C before analysis. The specific surface area, S_BET_, was calculated using the linear part of the BET plot. The isotherms were obtained at −196°C. The mesoporous size distributions were obtained by applying the Barrett-Joyner-Halenda (BJH) equation to the desorption branch of isotherms. The estimation of the total pore volume was made from the N_2_ uptake at a P/P0 value of 0.995.

A L230 Beckman-Coulter light scattering laser was employed in order to measure the particle size of the catalyst before and after catalytic test. Ethanol was used as a solvent in order to avoid the agglomeration of particles during the analysis.

In order to study the nature of the carbonaceous species deposited on the catalyst after catalytic tests, cross-polarization (CP), and magic angle spinning (MAS) ^13^C-NMR (CPMAS ^13^C-NMR) spectroscopy was carried out. The spectroscopy experiments were conducted on Bruker ASX400 (9.4 T) spectrometers operating at frequencies 100.6 MHz and using a 4 mm rotor probe. The rotor spinning rate was 10 kHz.

## Results and Discussion

### Hydrodynamic Study

A hydrodynamic study was first carried out to check the fluidization quality, which has a direct impact on the catalytic performances. The study was performed at 275°C, corresponding thus to the reaction temperature. Hydrodynamic parameters such as the quality of fluidization and minimum fluidization velocity were determined. As mentioned before, an attrition study of the catalyst was also carried out.

**Figure 3:**
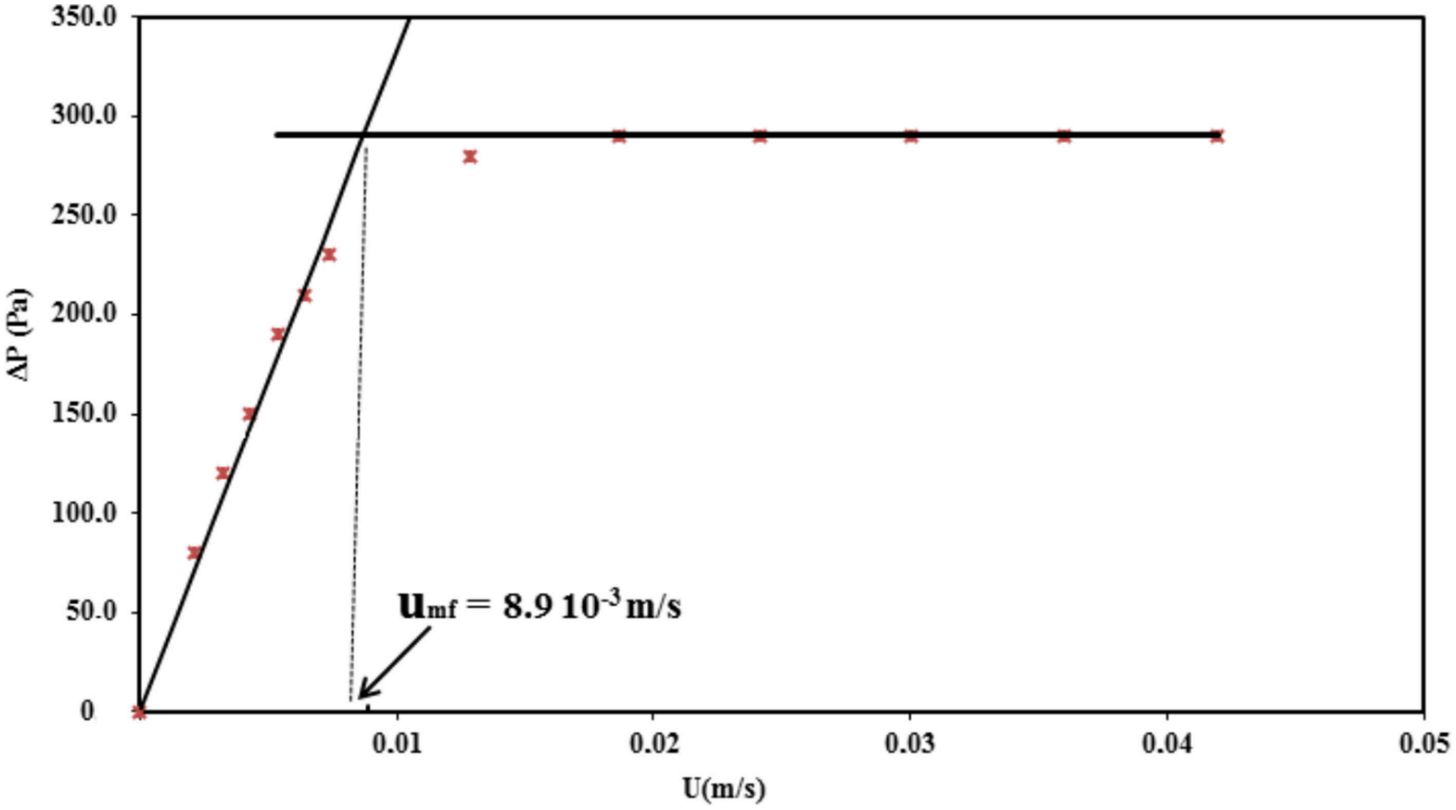
Pressure drop as a function of the gas velocity at 275°C using the gas velocity decreasing branch.

In order to avoid the hysteresis phenomenon during the measurement of the pressure drop (Δ*P*) with the increasing velocity of the gas, all the measurements of the Δ*P* curves were carried out while decreasing the superficial gas velocity (*U*) (Figure 3). This curve enables determining the minimum fluidization velocity *u*_*mf*_ by applying the standardized Richardson's method (Richardson et al., 1971) *u*_*mf*_ is thus located at the intersection of the sloping line corresponding to the linear increase of Δ*P* in the fixed bed and of the horizontal line corresponding to the fluidized regime (Figure 3). The as-determined minimum fluidization velocity for the catalyst was 8.9.10^−3^ m/s.

Using Figure 3, it was also possible to evaluate the quantity of solid participating in the fluidization, described by the so-called “fluidization quality,” further noted as α and defined as follows:

(5) $$\begin{array}{l} \alpha =\frac{\;{\Delta P}_{experimental}}{{\left(m.g/A\right)}_{\;}} \end{array}$$

Where Δ*P*_*experimental*_ corresponds to the Δ*P* measured when the fluidization regime is reached (i.e., the horizontal line on Figure 3), *m* is the mass of catalyst loaded in the reactor (96 g), *g* the standard gravity (9.81 m.s^−2^), and *A* the cross-section of the catalytic bed (1.96.10^−3^ m^2^ corresponding to an internal diameter of 5 cm).

The fluidization quality was 83%, meaning that more than 80% of the catalyst participated in the fluidization, the remainder being probably located in dead zones of the reactor. Such a high fluidization quality in a small-scale fluidized bed reactor can be considered as very good, offering reassurance for the appropriate design of the TZFBR used herein.

### Attrition Test for Catalysts

In a fluidized bed, the attrition of catalyst particles is most of the time unavoidable. In general, the attrition phenomenon can be classified into two main types: particles' fragmentation and surface abrasion. These phenomena could lead to a certain loss in the active phase fraction located on the outer surface of the particles and thus to a decrease in the catalytic performances. In order to verify the mechanical stability of the particles, an attrition experiment was carried out in the fluidized bed. The TZFBR was loaded with 96 g of fresh catalyst and then fluidized during 44 h with air at 275°C, without any glycerol feed, and using a gas velocity roughly twice as much as the minimum fluidization velocity *u*_*mf*_ determined previously (namely 18.6.10^−3^ m/s). Note that it also corresponded to the velocity selected for the subsequent catalytic tests. In those conditions, the height of the fluidized bed was around 15 cm. For this experiment, the cool-trap system (Figure 2) was replaced by a cyclone so as to recover the small particles possibly elutriated out by the gas during the test. The particles of the catalyst were recovered in the TZFBR after the attrition test and subsequently analyzed by light scattering laser, SEM, nitrogen physisorption, Raman spectroscopy and elemental analysis (ICP-MS).

**Figure 4:**
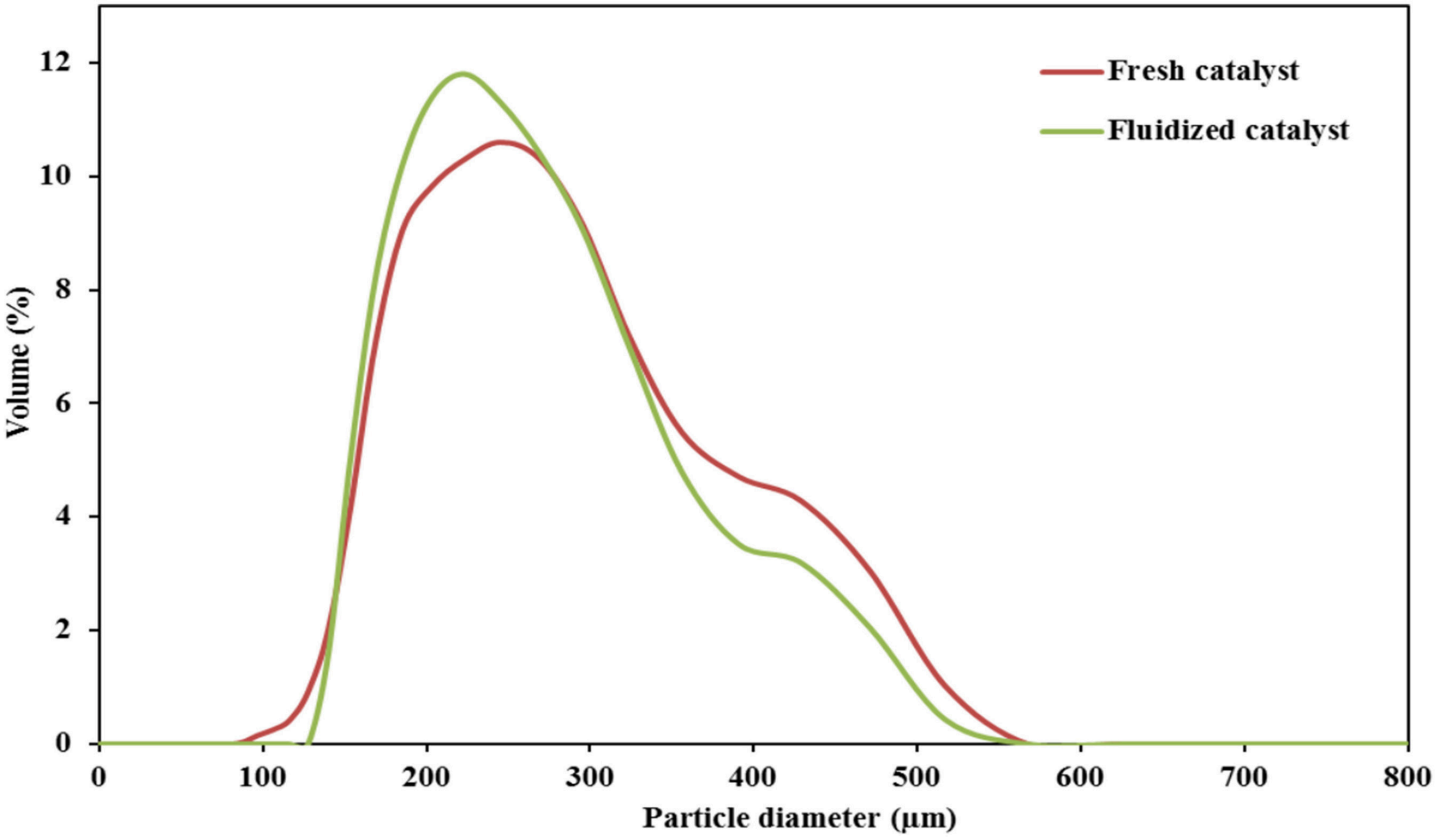
Particles' size distribution of the fresh catalyst and after a 44 h attrition test in the TZBR at 275°C.

In Figure 4, it can be noticed that the size distribution curve of the fluidized catalyst is slightly less broad than that of the fresh one. This indicates a slight abrasion of the catalyst after the attrition test. The recovered mass of the catalyst was 94 g after the attrition test (corresponding to 98% of the initial loading). No particles were collected in the cyclone. The loss of 2 wt.% is within the experimental accuracy and thus does not constitute strong evidence of the elutriation of fine particles.

**Figure 5:**
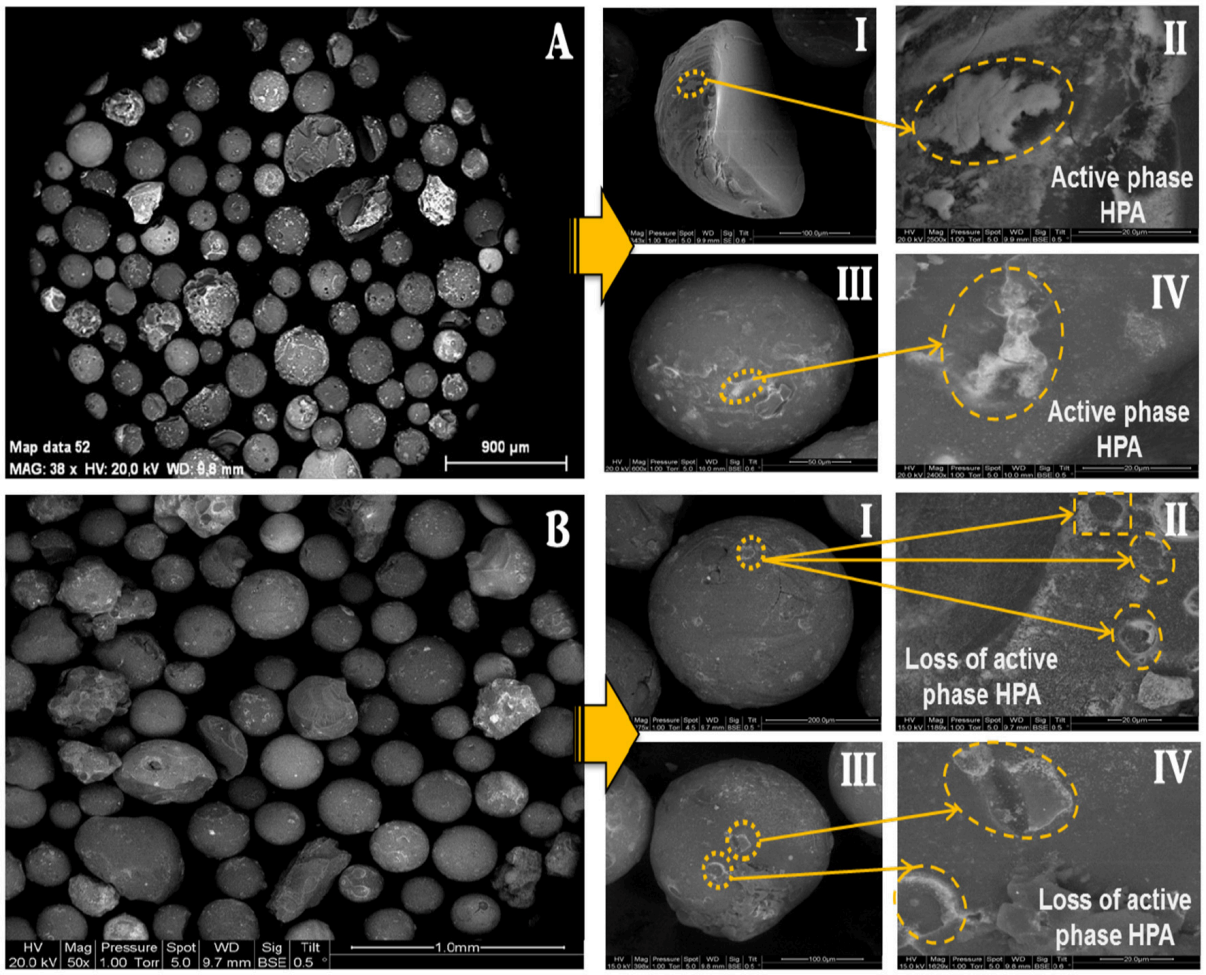
Scanning Electron Micrographs of **(A)** the fresh catalyst, and **(B)** the catalyst after attrition test (after 44 h of fluidization at 275°C).

The SEM images of the catalyst particles before and after the attrition test are presented in Figure 5. It was observed that the fluidized catalyst substantially keeps the same morphology as that of the fresh one, meaning that no fragmentation occurred during the fluidization process. However, it seems that the active phase deposited on the external surface of the support in plate-like spots (Figures 5 A-II,IV) was somewhat eroded after 44 h of fluidization, with some tear-like damage marks detected on the surface of the particles (Figures 5 B-II,IV).

Similarly, the comparison of the physical and textural properties (Table 2) such as the particle diameter, the specific surface area and the pore volume of the catalyst before and after 44 h of fluidization (attrition test) did not show any significant change. Only a slight decrease in the mean particle diameter determined by light scattering granulometry from 245 to 233 μm was observed after fluidization. One can assume that this resulted from the abrasion phenomenon.

**Table 2:** Physical and textural properties of the catalyst before and after the attrition test.

**Parameter**	**Fresh catalyst**	**Catalyst after fluidization**
Particle diameter	245 μm	233 μm
Surface area	230 m^2^/g	230 m^2^/g
Pore volume	0.92 cm^3^/g	1.01 cm^3^/g

**Figure 6:**
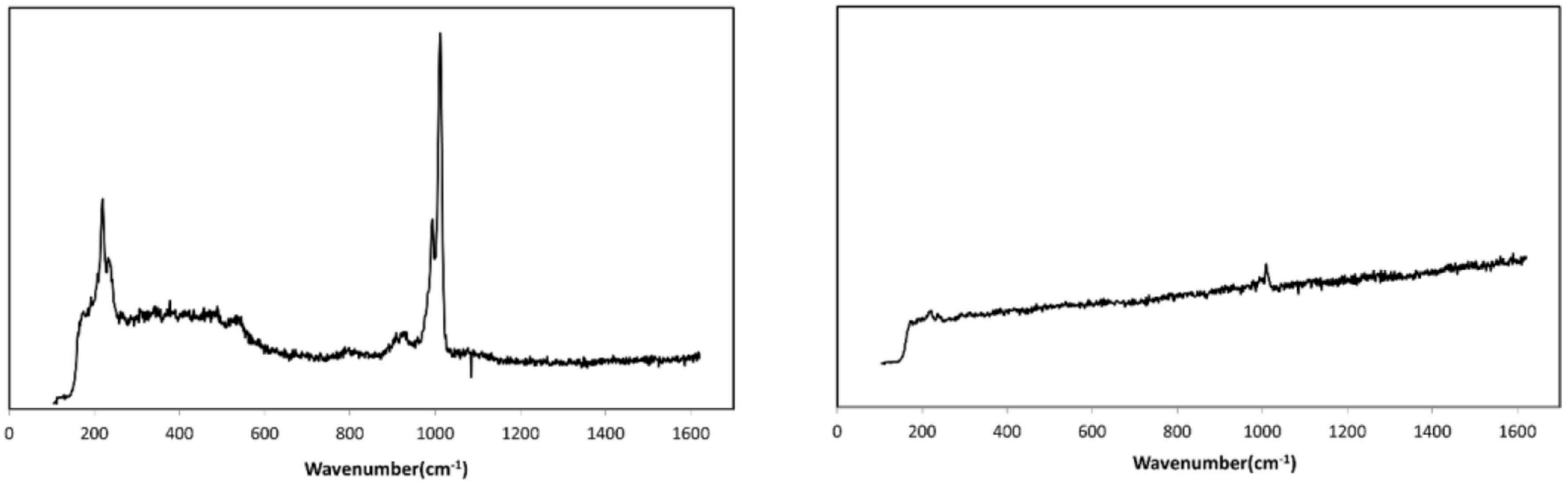
Raman spectra of fresh **(Left)** and fluidized catalyst particle **(Right)** (attrition test: 44 h of fluidization).

In order to evidence the hypothesis of an abrasion, the surface of the catalyst was analyzed by Raman spectroscopy. In fact, the collision and friction between particles during the fluidization could lead to a loss of the active phase (H_3_PW_12_O_40_) by abrasion from the catalyst surface, which would be easily evidenced due to the specific Raman bands of phosphotungstic acid. Raman spectra were recorded for several particles of fresh and used catalysts for comparison. Figure 6 shows the exemplary Raman spectra for a fresh (left) and a used catalyst particle (right). It is very clear that the fresh catalyst exhibits the typical bands of H_3_PW_12_O_40_, notably the W=O stretching vibration at ≈ 1,000 cm^−1^ whereas for the used catalyst almost no active phase was detected on the outer surface (Thouvenot et al., 1984). This evidenced clearly the abrasion of the HPA from the external surface of the catalyst. Nevertheless, as the Raman technique is a surface-sensitive technique, one cannot draw conclusions regarding the behavior of the active phase present in the pores of the catalyst.

Therefore, in order to complete the Raman study and to estimate the loss of active phase during the attrition test, ICP-MS analyses of the fresh and used catalysts were done. From Table 3, where the calculated amounts of both HPA and support are reported, it can be noted that the compositions in HPA for the fresh and the used catalysts were close to the theoretical weight percentages expected (19 and 18 ± 1 vs. 20 wt.% for the fresh and used samples, respectively). It can therefore be concluded that the catalyst did not undergo a significant loss of active phase after 44 h of fluidization. Nevertheless, as said above, the SEM analysis showed a slight abrasion of the active phase on the external surface of the catalyst. It is believed that the particles produced by the abrasion are very fine and elutriated by the gas stream from the reactor and cannot be trapped in the cyclone, which was not sufficiently efficient to handle such fine particles. Consequently, from the analysis of physical and textural properties as well as the Raman, SEM and ICP-MS analysis, it may be asserted that the catalyst particles undergo a slight loss of active phase by mechanical abrasion during the fluidization. However, the loss of active phase remains limited to the catalyst's external surface and is not significant in comparison to the amount of active phase, which is present in the internal porosity of the silica support.

**Table 3:** Weight percentages of active phase and support obtained by ICP-MS.

**Compound**	**Fresh catalyst**	**Fluidized catalyst**
H_3_PW_12_O_40_	19 ± 1 wt.%	18 ± 1 wt.%
SiO_2_	81 ± 1 wt.%	82 ± 1 wt.%

### Catalytic Tests

The stability of the catalytic performances in a TZFBR depends on the balance between the rates of the coking of the catalyst under dehydration reaction conditions and of the regeneration of the catalyst in the presence of oxygen in the fluidizing gas. If the regeneration is not sufficiently efficient, coke will accumulate on the surface and in the pores of the catalyst, causing its progressive deactivation. If the regeneration is effective, the formation of coke and the oxidative removal are balanced, avoiding thus the over-accumulation of carbonaceous species. On the other hand, if too much oxygen is present in the reaction zone, it will react with glycerol, acrolein, or others to yield undesired products. Hence, the tuning of the operating conditions of the TZFBR must be very precise to find a good compromise. In order to better control this aspect, two parameters were studied in this work: (i) the relative volumes of the regeneration and reaction zones, and (ii) the oxygen/glycerol molar ratio. The total height (and consequently volume) of the fluidized-bed is constant at a given set of operating conditions (about 15 cm height in our case) but, as mentioned before, the respective volumes of the regeneration and reaction zones can be modified by adjusting the vertical position of the injection nozzle of the glycerol feed. Moreover, the oxygen/glycerol ratio can be tuned using different amounts of air mixed with the nitrogen flow in the fluidization gas.

#### Influence of the Volumes of the Reaction and Regeneration Zones

In order to observe the influence of the relative volumes of the regeneration and reaction zones, the vertical position of the glycerol injection nozzle was varied. More precisely, the glycerol feed was injected at 5 and 8 cm above the gas distributor. Considering that the fluidized bed's total height was 15 cm, the regeneration and reaction zones' heights were 5 and 10 cm, respectively, in the first case, and 8 and 7 cm, respectively, in the second configuration. In other words, the reaction zone was twice the size of the regeneration zone in the first case (*V*_*reaction*_/*V*_*regeneration*_ = 2) and roughly the same size in the second configuration (*V*_*reaction*_/*V*_*regeneration*_ = 1).

**Figure 7:**
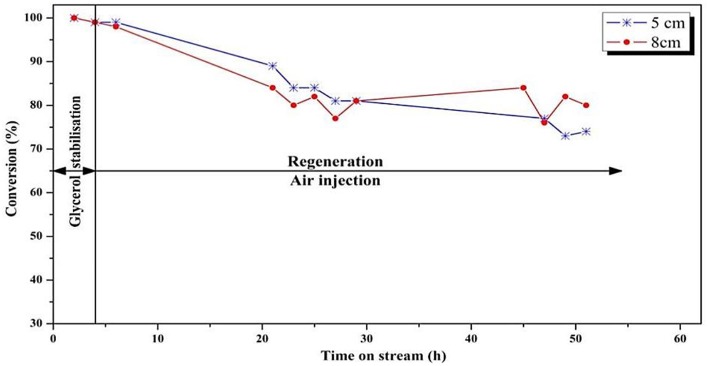
Influence of position of the feed injection in the fluidized-bed on the conversion of glycerol (*T* = 275°C, O_2_/Gly = 0.4, gas velocity = 1.86.10^−2^ m/s). Five centimeters correspond to *V*_*reaction*_/*V*_*regeneration*_ = 2 whereas 8 cm correspond to *V*_*reaction*_/*V*_*regeneration*_ = 1.

**Figure 8:**
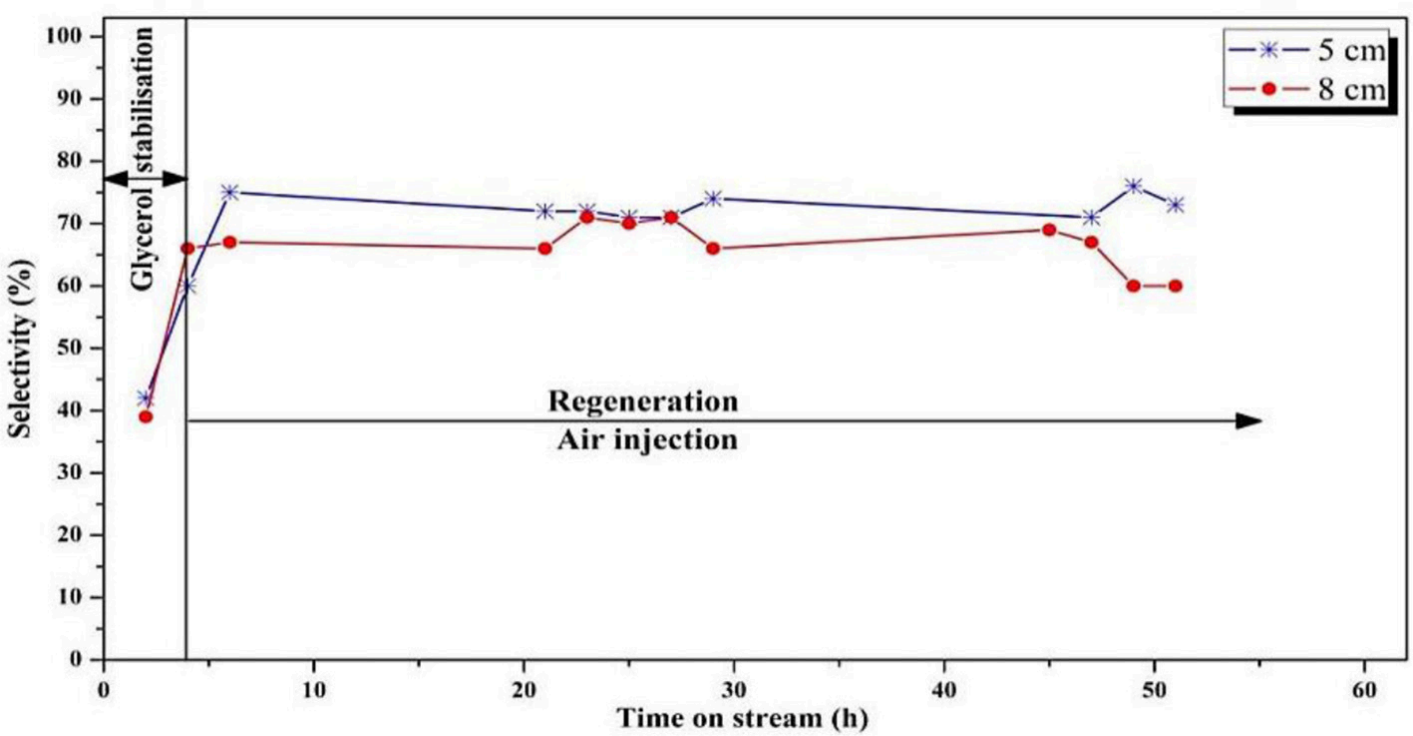
Influence of position of the feed injection nozzle in the fluidized bed on the selectivity to acrolein (*T* = 275°C, O_2_/Gly = 0.4, gas velocity = 1.86.10^−2^ m/s). Five centimeters correspond to *V*_*reaction*_/*V*_*regeneration*_ = 2 whereas 8 cm correspond to *V*_*reaction*_/*V*_*regeneration*_ = 1.

The glycerol conversion and acrolein selectivity vs. time on stream are shown in Figures 7, 8, respectively. Figure 7 shows that a larger regeneration zone led to a lower conversion during the first 20 h on stream, which then remained rather constant until the end of the test (50 h), meaning that the catalyst reached a steady state after 20 h of reaction. On the other hand, with a smaller regeneration zone, it seems that the catalyst showed a constant deactivation, without reaching a steady-state. With respect to the accuracy of ± 2% in the conversion measurement, one can state that the difference observed between both experiments was significant, meaning that a too-small regeneration zone (*V*_*reaction*_/*V*_*regeneration*_ = 2) did not enable continuous regeneration of the coked catalyst.

Concerning the acrolein selectivity (Figure 8), quite similar values (65–70%) were observed in both experiments. Only small amounts of byproducts such as acetaldehyde (2%), acetone (1%), propionaldehyde (1%), and hydroxyacetone (3%) were observed. Carbon balances of 84 and 80% for the smaller and the larger regeneration zone were, respectively, observed. The incondensable CO and CO_2_ gas quantities could not be measured because of dilution that was too high in the fluidization gas outlet stream, and are hence not included in the carbon balance, explaining the low values.

#### Influence of the Oxygen/Glycerol Molar Ratio

**Figure 9:**
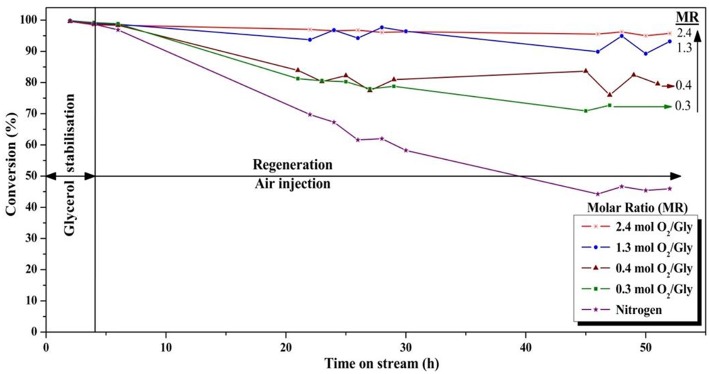
Influence of the oxygen/glycerol molar ratio on the conversion of glycerol (*T* = 275°C, position of the feed injection nozzle = 8 cm above the gas distributor, gas velocity = 1.86.10^−2^ m/s).

**Figure 10:**
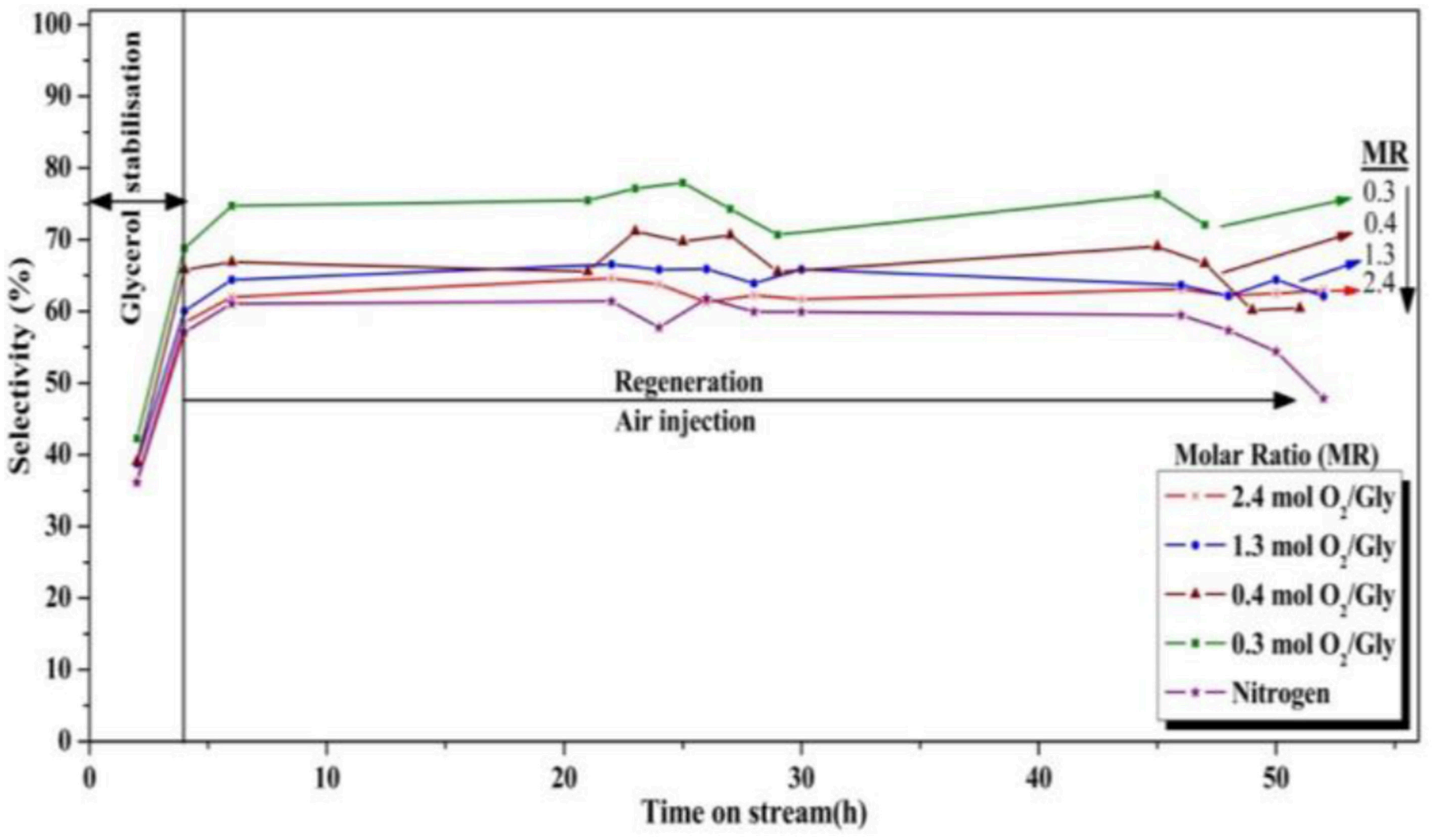
Influence of the oxygen/glycerol molar ratio on the selectivity to acrolein (*T* = 275°C, position of the feed injection nozzle = 8 cm above the gas distributor, gas velocity = 1.86 10^−2^ m/s).

Catalytic tests were performed using various oxygen/glycerol molar ratios, namely 0 (only nitrogen is injected in the fluidisation gas), 0.3, 0.4, 1.3, and 2.4 at 275°C with *V*_*reaction*_/*V*_*regeneration*_ = 1 (injection nozzle at 8 cm above the gas distributor). The results are given in Figures 9, 10 for glycerol conversion and acrolein selectivity, respectively.

The use of higher quantities of oxygen in the feed was clearly found to be favorable for the glycerol conversion. With an oxygen/glycerol molar ratio of 2.4, a high and stable conversion of ca. 96% was observed during the 52 h of the experiment, which is remarkable. The conversion decreased in the same order as that of the oxygen-to-glycerol molar ratio, with 75, 80, and 90% after 52 h on stream for molar ratios of 0.3, 0.4, and 1.3, respectively. It is worth mentioning that for a ratio of 1.3, a high conversion of glycerol around 95% was observed during the first 30 h, with a slight decrease of 5 points after 52 h. When no oxygen was injected, a fast deactivation of the catalyst occurred as expected, and no more than 45% of the fed glycerol were converted after 52 h under stream.

On the other hand, high O_2_/glycerol molar ratios led to a decrease in acrolein selectivity (Figure 10). While a high selectivity to acrolein (≈ 75%) was observed at a molar ratio of 0.3, it was only 60% when using a molar ratio of 2.4. This decrease in acrolein selectivity can be explained by the decomposition of the as-formed acrolein in oxygen excess. In fact, based on a rough calculation, an O_2_/Gly ratio of 0.3 should roughly allow the oxidization of carbonaceous species corresponding to a carbon selectivity of 15%. Hence, when using larger amounts of oxygen for the regeneration, one can suppose that significant parts of the introduced oxygen are not consumed in the regeneration zone for the combustion of the deposited coke, whereby the unreacted oxygen can interact with glycerol or acrolein in the reaction zone, causing notably total oxidation to CO_x_. Next to acrolein, only small amounts of condensable byproducts such as acetaldehyde (3%), propionaldehyde (1%), and acrylic acid (1%) were observed.

Surprisingly, the selectivity to acrolein was found to be very low in total absence of oxygen in the feed. The surface of the catalyst was probably already too covered by coke after the 4-h stabilization period, preventing the reactant from accessing the selective active sites where acrolein is formed by glycerol conversion. Moreover, the quality of fluidization can be significantly affected for strongly coked catalyst.

Nevertheless, it is noteworthy that in the best conditions tested in the present study (O_2_/Gly = 1.3 and 2.4), a stable yield in acrolein of around 60–65% could be maintained, which would have been impossible in a fixed-bed reactor without oxygen injection.

**Figure 11:**
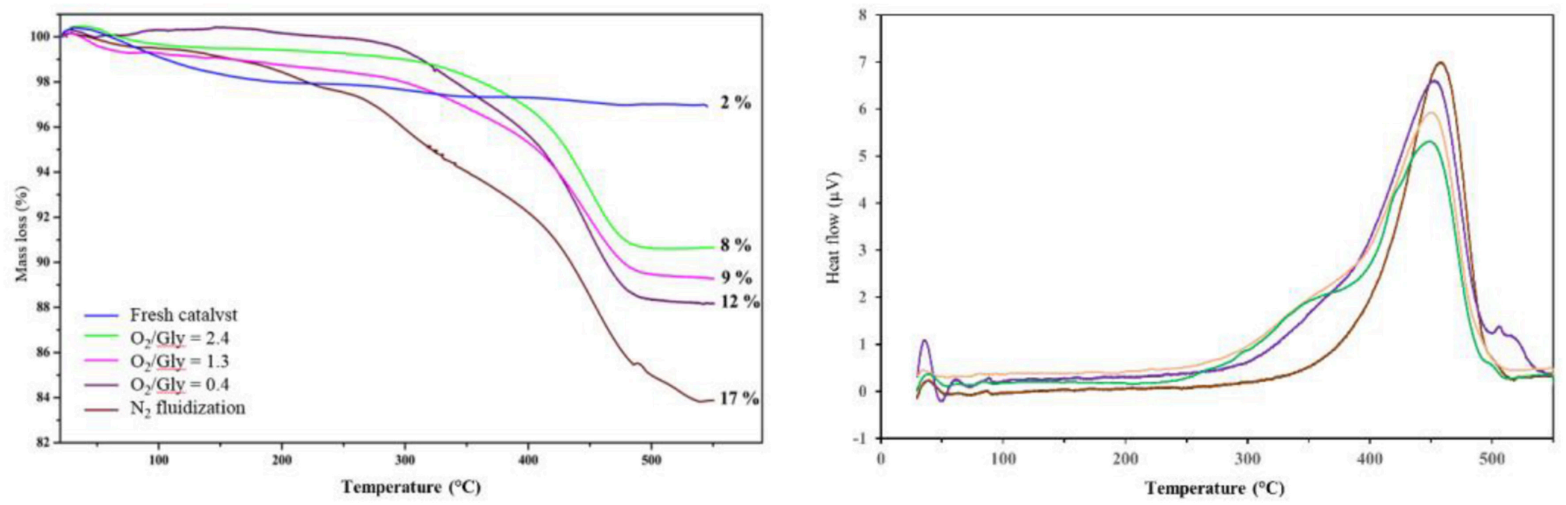
Influence of the oxygen/glycerol molar ratio on the amount of coke after test, determined by TGA **(Left)** analysis and DSC **(Right)**.

**Figure 12:**
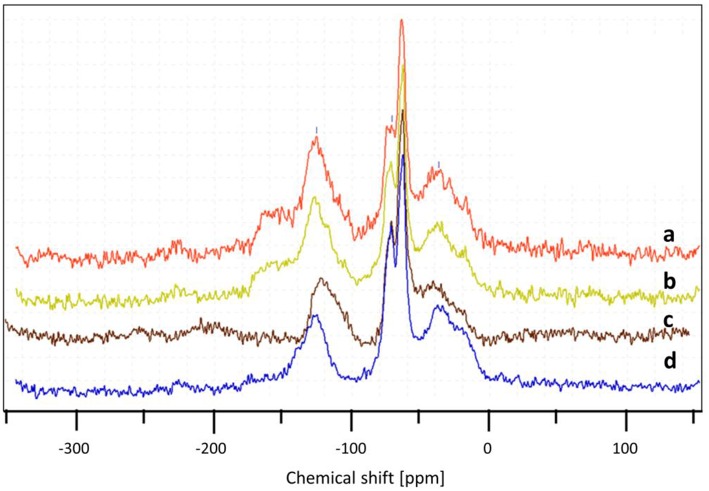
Influence of the oxygen/glycerol molar ratio on the nature of carbonaceous species after test, determined by ^13^C CP MAS NMR for (O_2_/Gly) = 2.4 **(a)**, 1.3 **(b)**, 0.4 **(c)**, and pure nitrogen **(d)**.

In order to check the influence of the O_2_/Gly ratio on the amount of coke formed on the catalyst, the particles recovered after the test were analyzed by TGA and DSC (Figure 11). The results showed the formation of carbonaceous species for all catalyst, but in different extents depending on the amount of oxygen injected in the TZFBR. In all cases, the loss of the carbonaceous species was accompanied by an exothermal peak, corresponding to the burning of the coke. Concerning the quantification, the amount of coke increased when decreasing the O_2_/Gly ratio from 8 wt.% (O_2_/Gly = 2.4) to 17 wt.% (no oxygen in the fluidization gas). Considering the stable performance observed for a O_2_/Gly ratio of 2.4, one can conclude that 8 wt.% of carbonaceous species are still acceptable for obtaining full conversion. In addition to the quantification of the deposed carbonaceous species, the latter were characterized using solid-state NMR on the ^13^C carbon with cross-polarization (CP) and magic-angle spinning (MAS). From the results (Figure 12), no significant change is observed in the nature of the carbonaceous species deposited on the catalyst under various molar ratios of oxygen or on the catalyst employed under pure nitrogen. This can be explained by the fact that ^13^C CP-MAS NMR is a non-quantitative technique. Nevertheless, one can note that the coke for all catalysts is basically composed by an aliphatic part (peaks around −37, −64, −71 ppm) and an aromatic part (peak around −125 ppm).

## Conclusion

The TZFBR concept was applied to the dehydration of glycerol to acrolein using a heteropolyacid-based supported catalyst. The catalyst was easily synthesized by wet impregnation of a commercial silica. The hydrodynamic behavior of the TZFBR shows good mixing, illustrated by the fluidization quality being superior to 80% at a reaction temperature of 275°C. The mechanical stability of the catalyst was controlled by performing an attrition test of the catalyst particles in the TZFBR at 275°C over 44 h in the absence of glycerol. The results show good mechanical resistance of the catalyst, whereby only a small quantity of active phase on its external surface was elutriated.

Concerning the catalytic results, the effect of the O_2_/glycerol ratio and of the size of the reaction/regeneration zone was studied, showing that a regeneration zone that was too small (*V*_*reaction*_/*V*_*regeneration*_ = 2) did not allow efficient catalyst regeneration. Concerning the O_2_/glycerol ratio, an inverse trend between conversion and selectivity was found: increasing the O_2_/glycerol ratio led to higher conversion (and lower coke deposit as shown by TGA analysis), but at the expense of the selectivity to acrolein, supposedly due to the presence of O_2_ in the reaction zone then burning glycerol and acrolein.

## Data Availability

All datasets generated for this study are included in the manuscript and/or the supplementary files.

## Author Contributions

RM: experiments, interpretation, writing of the first draft; SP, BK, NF, and FD: design of the study, experiments design, supervision, interpretation, and writing; VB-B and PR: design of the study, supervision, interpretation, and writing. All authors contributed to manuscript revision, read, and approved the submitted version.

### Conflict of Interest Statement

VB-B and PR are employed by ADISSEO Company. The remaining authors declare that the research was conducted in the absence of any commercial or financial relationships that could be construed as a potential conflict of interest.
